# Comparison of bacterial communities and antibiotic resistance genes in oxidation ditches and membrane bioreactors

**DOI:** 10.1038/s41598-021-88335-z

**Published:** 2021-04-26

**Authors:** Lifang Luo, Junqin Yao, Weiguo Liu, Lixin Yang, Hailong Li, Ming Liang, Hui Ma, Ziteng Liu, Yinguang Chen

**Affiliations:** 1 College of Resource and Environmental Science, Xinjiang University, No. 666 Shengli Road, Tianshan District, Urumqi, China; 2 Urumqi Hexi Water Supply Co. Ltd., Urumqi, 830000 Xinjiang China; 3 College of Environmental Science and Engineering, Tongji University, 1239 Siping Road, Shanghai, China

**Keywords:** Microbiology, Environmental sciences

## Abstract

Oxidation ditches (ODs) and membrane bioreactors (MBRs) are widely used in wastewater treatment plants (WWTPs) with bacteria and antibiotic resistance genes (ARGs) running through the whole system. In this study, metagenomic sequencing was used to compare the bacterial communities and ARGs in the OD and MBR systems, which received the same influent in a WWTP located in Xinjiang, China. The results showed that the removal efficiency of pollutants by the MBR process was better than that by the OD process. The composition and the relative abundance of bacteria in activated sludge were similar at the phylum and genus levels and were not affected by process type. Multidrug, fluoroquinolones and peptides were the main ARG types for the two processes, with *macB* being the main ARG subtype, and the relative abundance of ARG subtypes in MBR effluent was much higher than that in the OD effluent. The mobile genetic elements (MGEs) in the activated sludge were mainly transposons (*tnpA*) and insertion sequences (ISs; *IS91*). These results provide a theoretical basis for process selection and controlling the spread of ARGs.

## Introduction

The activated sludge process is currently the most widely used wastewater treatment technology. Oxidation ditches (ODs) and membrane bioreactors (MBRs), which are the two main activated sludge processes, utilize different "sludge-water" separation methods. Specifically, the secondary sedimentation tank following the OD relies on gravity sedimentation, while the MBR relies on filtration and interception via the membrane^1^. These systems have unique artificial microbial ecosystems that characteristically harbor high microbial diversity and a large number of microorganisms, especially bacteria carrying various types of antibiotic resistance genes (ARGs), which can also drive changes in ARGs^2,3^. As an emerging type of biological pollutant, ARGs can persist, transfer and spread within different environmental media, causing serious harm to human health^4^.

The pollutant removal performance and stable operation of the treatment system depend on the microorganisms, with bacteria playing a key role^5^. At present, many studies have explored the bacterial communities in OD and MBR systems. These studies showed that the bacterial community members at the phylum level in the two systems are similar, with Proteobacteria and Bacteroides generally dominating^6,7^. However, there were some differences at the genus level. The OD system produces alternating anoxic and aerobic conditions in space and time, enabling nitrification and denitrification to be carried out simultaneously^8^. Subsequently, *Nitrospira*^9,10^, *Nitrosococcus*^10^, *Nitrosomonas*^8^, *Thauera*^8,9^, *Arcobacter*^8^ and *Zoogloea*^8^ were detected as the most abundant nitrogen removal bacteria. *Dechloromonas*, a key phosphate-accumulating organisms (PAOs), has been detected with high abundance in ODs. Compared with ODs, MBRs have a longer sludge residence time (SRT), which provides favorable living conditions for some slow-growing microorganisms, especially the autotrophic nitrifying bacteria *Nitrosomonas*^11^ and *Nitrospira*^1,11,12^. Moreover, membrane interception results in richer microbial diversity and higher microbial biomass. Accordingly, *Zoogloea*^1^, *Flavobacterium*^7^, *Thauera*^1,7^, *Comamonas*^7^, *Haliscomenobacter*^12^ and *Rhodobacter*^12^ are considered dominant, and the PAOs *Tetrasphaera* and *Accumulibacter* are frequently detected in MBRs^13,14^. These abundant bacterial genera are widely distributed in the treatment system and are responsible for effectively removing nitrogen, phosphorus and other pollutants, as well as ensuring stable effluent quality^7,12,13^.

Bacteria are generally considered to be carriers of ARGs^15^. Some bacteria that remove nitrogen and phosphorus, such as *Nitrosomonas*^16,17^, *Nitrospira*^16^, *Pseudomonas*^3,16^, *Acidovorax*^18^, *Comamonas*^18^, *Dechloromonas*^17^ and *Accumulibacter*^17^, have been widely confirmed as hosts of various ARGs, which makes it possible for ARGs to be widely distributed in biological sewage treatment systems^19^. To date, various types of ARGs have been detected in wastewater treatment plants (WWTPs) from many regions. For instance, WWTPs from Beijing (China)^17^, Shanghai (China)^16^, Hong Kong (China)^20^, Taiwan (China)^21^ and Michigan (USA)^22^ harbor many types of ARGs, among which it is generally believed that multidrug-, tetracycline- and sulfonamide-resistant genes dominate. Metagenomic sequencing was used to identify ARGs in activated sludge from 4 WWTPs in China using anaerobic/anoxic/oxic (A^2^O) + MBR and A^2^O processes. The results showed that the diversity of ARG subtypes was similar, mainly *EF-TU*, *rpoB* and *rpsL*^16^. Likewise, a qPCR-based study of ARGs in the activated sludge of 16 WWTPs using OD, A^2^O, and sequencing batch reactor (SBR) processes in 9 different areas of China found that the dominant ARG subtypes of the different WWTPs were similar, all of which were the *aacC2* subtype^23^. ARGs can facilitate horizontal gene transfer between bacterial strains of the same and different species through mobile genetic elements (MGEs), such as integrons, transposons and plasmids, which presents a hidden risk to public health safety^24^.

Although there are many studies describing the bacteria and ARGs from different processes, there are few studies comparing the bacteria and ARGs of different processes under the same influent conditions. Metagenomic sequencing targets the DNA of all microorganisms within a given environment, which makes up for the deficiencies of amplicon-based analysis; thus, it is useful for broad-spectrum ARG screening^17^. In this study, metagenomic analysis was used to compare the bacterial communities and ARGs in the OD and MBR systems. These results provide a theoretical reference for the comparison and selection of processes and show more information on the distribution of bacterial communities and ARGs in WWTPs. Moreover, this study offers insight for further controlling the prevalence of ARGs.

## Results

### Treatment performance of the WWTP

The quality of the incoming and outgoing water from the WWTP and the treatment performance are shown in Table 1. The average ratio of biochemical oxygen demand (BOD_5_) to chemical oxygen demand (COD) was 0.45 in the influent, indicating that the biochemical performance of the influent was good. The average ratios of BOD_5_ to total nitrogen (TN) and total phosphorus (TP) were 3.75 and 30.69, respectively. Thus, both treatment systems can operate efficiently and stably, and the treatment effects meet the design standards. Differences in the systems resulted in higher removal rates of NH_4_^+^-N and TN in the OD than in the MBR and higher removal rates of COD, BOD_5_, SS, and TP in the MBR than in the OD.

**Table 1:** Removal of contaminants by the OD and MBR processes.

Indicator	Influent (mg/L)	OD		MBR	
		Effluent (mg/L)	Removal efficiency (%)	Effluent (mg/L)	Removal efficiency (%)
COD	246–1012	10–31	82.0–99.0	8–28	85.1–99.7
BOD_5_	71–589	1–6	85.9–99.1	1–5	91.1–99.9
SS	94–520	1–10	96.0–99.2	1–7	96.8–99.8
NH_4_^+^-N	19.11–68.35	0.89–1.23	84.5–99.8	1.01–1.39	69.0–99.0
TN	32.10–94.32	1.93–13.5	62.0–96.8	3.12–14.78	46.1–91.6
TP	2.51–12.39	0.23–0.35	50.6–99.0	0.10–0.26	67.8–99.9

### Comparison of bacterial communities

**Figure 1:**
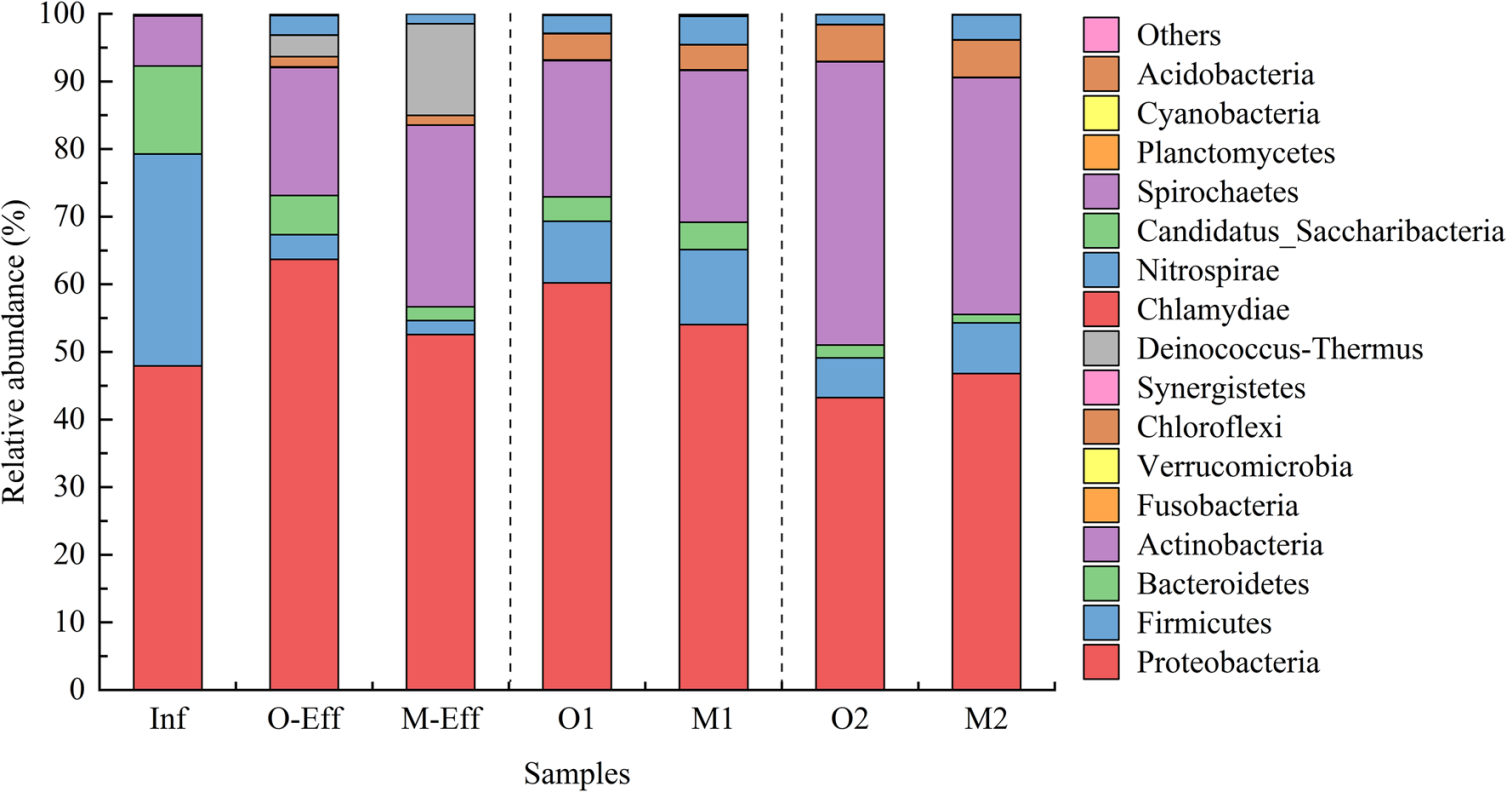
Community structure at the phylum level in the OD and MBR systems.

A total of 29 bacterial phyla were detected in all samples, of which Proteobacteria (43.24–63.69%) was the most abundant bacterial phylum, followed by Actinobacteria (7.42–41.88%), Firmicutes (2.07–31.32%), Bacteroidetes (1.23–13.01%), Chloroflexi (0.02–5.40%), and Nitrospirae (0.01–4.17%) (Fig. 1).

**Figure 2:**
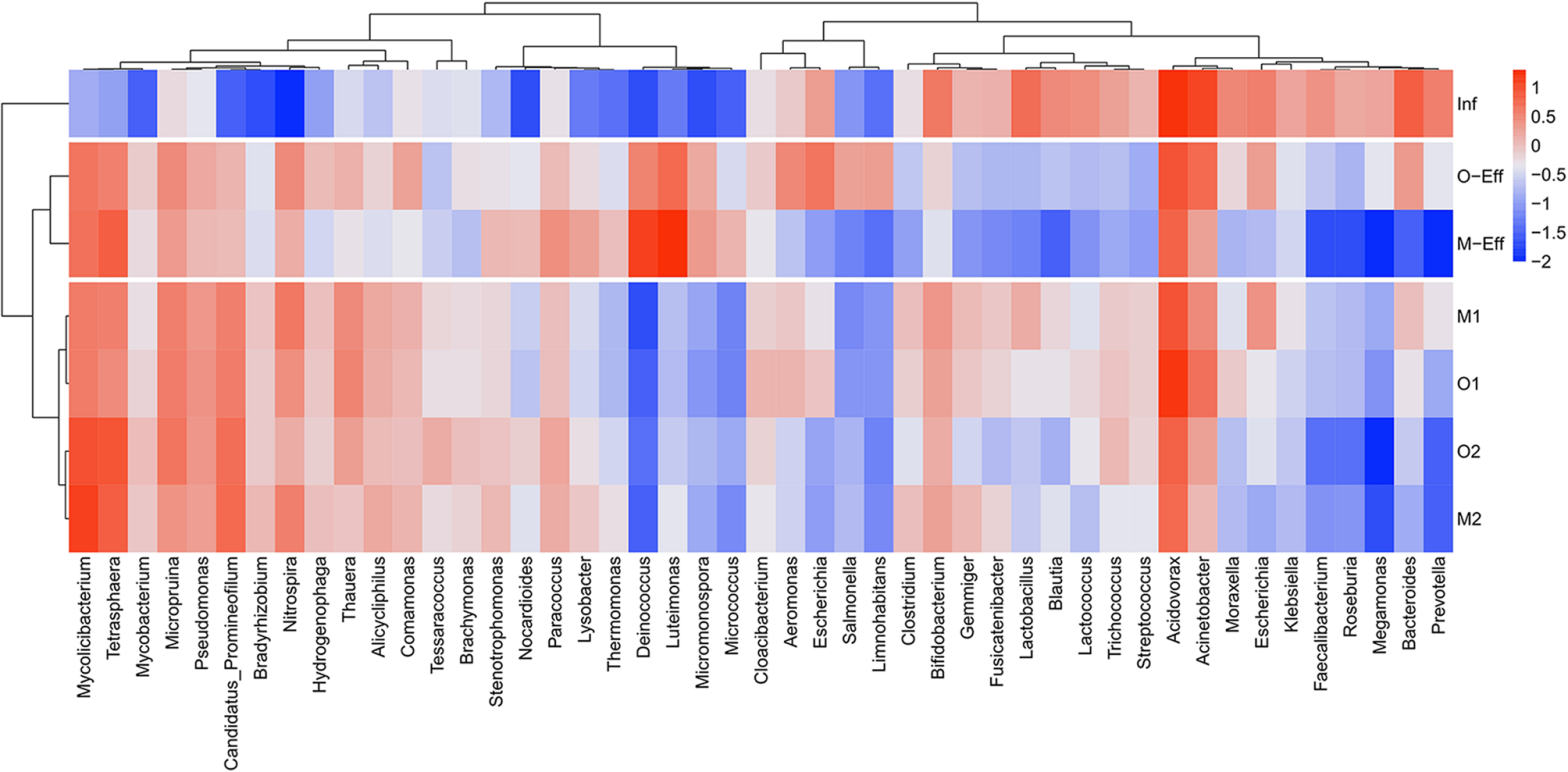
Community structure at the genus level in the OD and MBR systems.

At the genus level, a total of 1566 bacterial genera were assayed in all samples, and 48 with relative abundances greater than 1% were detected in at least one sample (Fig. 2). The predominant bacteria in sample Inf were *Acidovorax* (17.55%) followed by *Acinetobacter* (11.96%), *Bacteroides* (6.97%), and *Lactobacillus* (5.13%). *Acidovorax* (8.95%) was the most abundant genus in sample O-Eff, and the other major bacteria were *Luteimonas* (5.91%), *Acinetobacter* (5.78%), *Mycolicibacterium* (4.70%), *Escherichia* (4.47%), and *Tetrasphaera* (3.63%). The M-Eff sample harbored mainly *Luteimonas* (20.01%) and *Deinococcus* (13.62%), and other abundant genera were *Tetrasphaera* (6.91%), *Acidovorax* (6.43%) and *Mycolicibacterium* (6.83%). There was an obvious difference in the distribution of bacteria in and out of wastewater, and the enrichment degree of bacteria in the effluent of OD was different from that of MBR.

In activated sludge samples, the predominant bacterial genera in samples O1 and M1 were *Acidovorax* (16.03% and 9.23%) followed by *Mycolicibacterium* (4.02% and 3.92%), *Candidatus_Promineofilum* (3.87% and 3.66%), *Micropruina* (3.75% and 3.59%), *Thauera* (3.23% and 3.89%), and *Nitrospira* (2.72% and 4.26%). In samples O2 and M2, *Mycolicibacterium* (10.02% and 13.12%) was the most abundant, and *Tetrasphaera* (9.06% and 9.06%), *Acidovorax* (6.73% and 6.73%), *Candidatus_Promineofilum* (5.31% and 5.50%), and *Micropruina* (4.37% and 2.54%) had higher abundances. Overall, the distribution of bacteria in OD was similar to that in MBR activated sludge.

**Figure 3:**
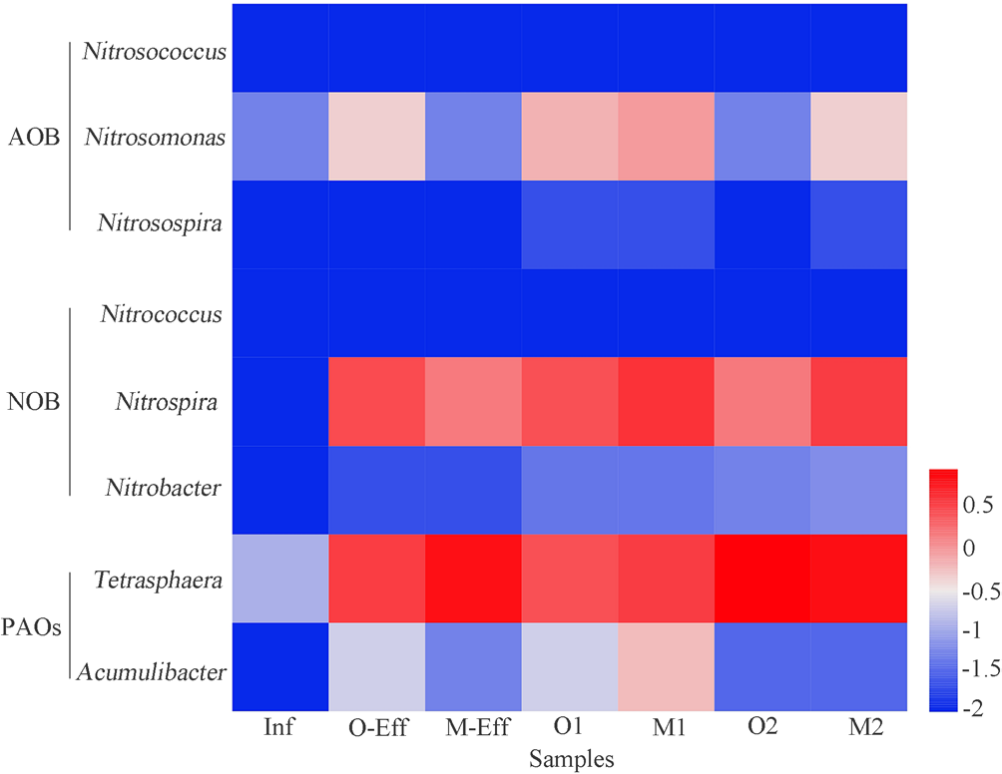
AOB, NOB, and PAOs distribution in all samples.

The detected species of bacteria related to nitrogen and phosphorus removal in activated sludge (Fig. 3) were ammonia-oxidizing bacteria (AOB; *Nitrosomonas* (0.04–0.90%) and *Nitrosospira* (0.01–0.02%); nitrite-oxidizing bacteria (NOB; *Nitrospira* (1.48–3.73%) and *Nitrobacter* (0.03–0.05%)); and PAOs (*Tetrasphaera* (2.79–9.06%) and *Accumulibacter* (0.18–0.58%)). Among them, the abundance of *Nitrosomonas* and *Nitrospira* in MBR activated sludge was distinctly higher than that in OD activated sludge.

### Comparison of ARGs

**Figure 4:**
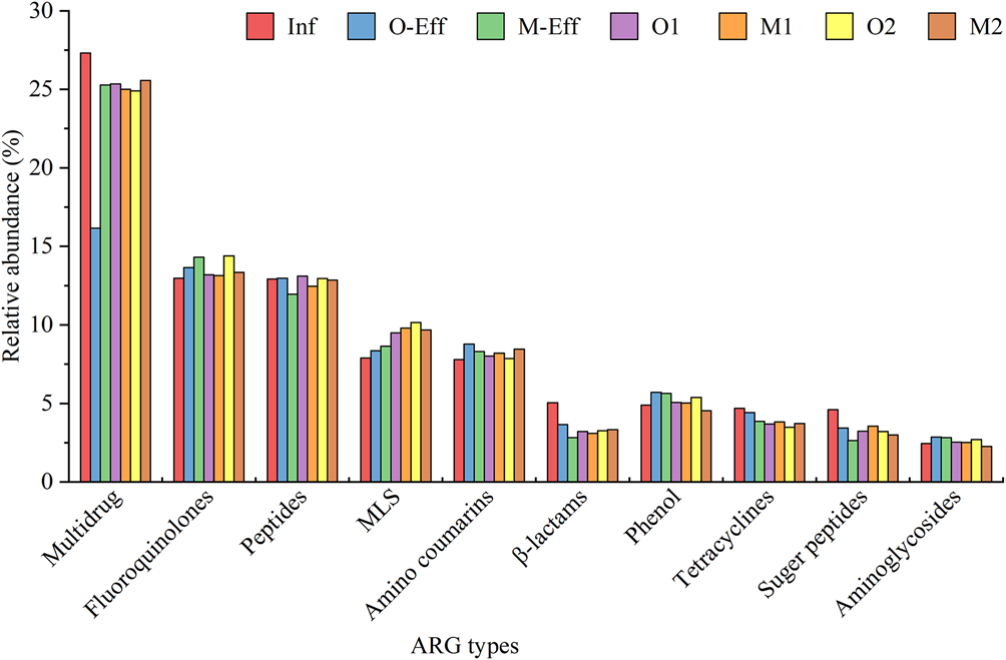
Distribution and relative abundance of the top 10 ARG types in all samples.

There were 10 ARG types with relatively high abundances in all samples (Fig. 4). Among them, the multidrug type was the most abundant, followed by fluoroquinolones and peptides, the sum of which was as high as 41.09–54.81%.

**Figure 5:**
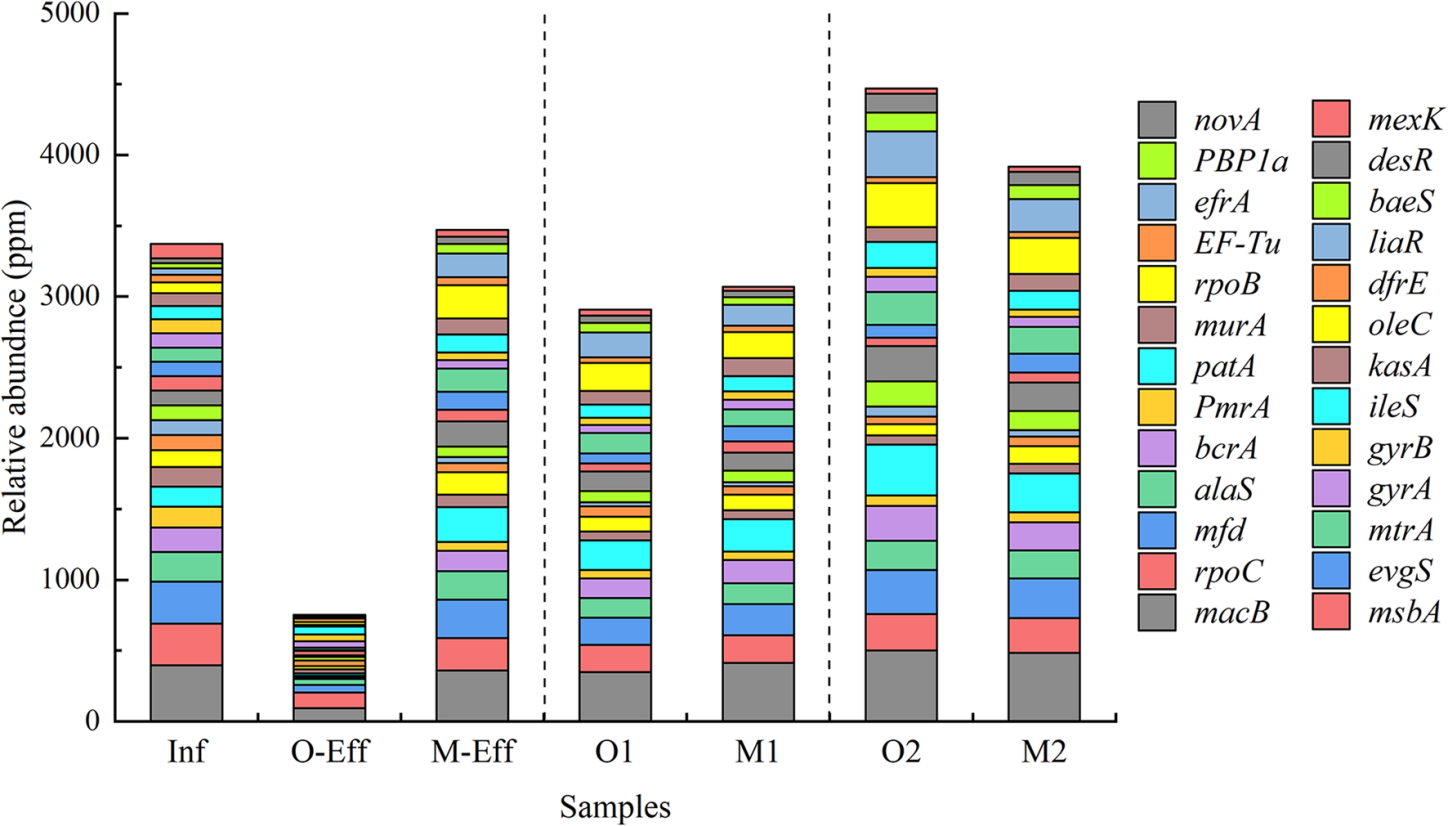
Distribution and relative abundance of the top 15 ARG subtypes in each sample.

Overall, 413–573 ARG subtypes were detected in all samples. Sample Inf had the most abundant subtypes (573), while sample O-Eff had the fewest subtypes (413). The total relative abundance was assigned to the top 15 ARG subtypes in all samples, ranging from 791.34 to 3774.37 ppm (Fig. 5), accounting for 50.23–53.23% of all ARGs, of which *macB* (95.16–500.71 ppm) had the highest abundance, followed by *rpoC* (100.99–296.52 ppm), *mfd* (52.61–312.24 ppm) and *patA* (19.21–359.20 ppm). The relative abundance of most ARGs in sample O-Eff was clearly lower than that in sample M-Eff and was not significantly different in activated sludge.

### Comparison of MGEs

**Figure 6:**
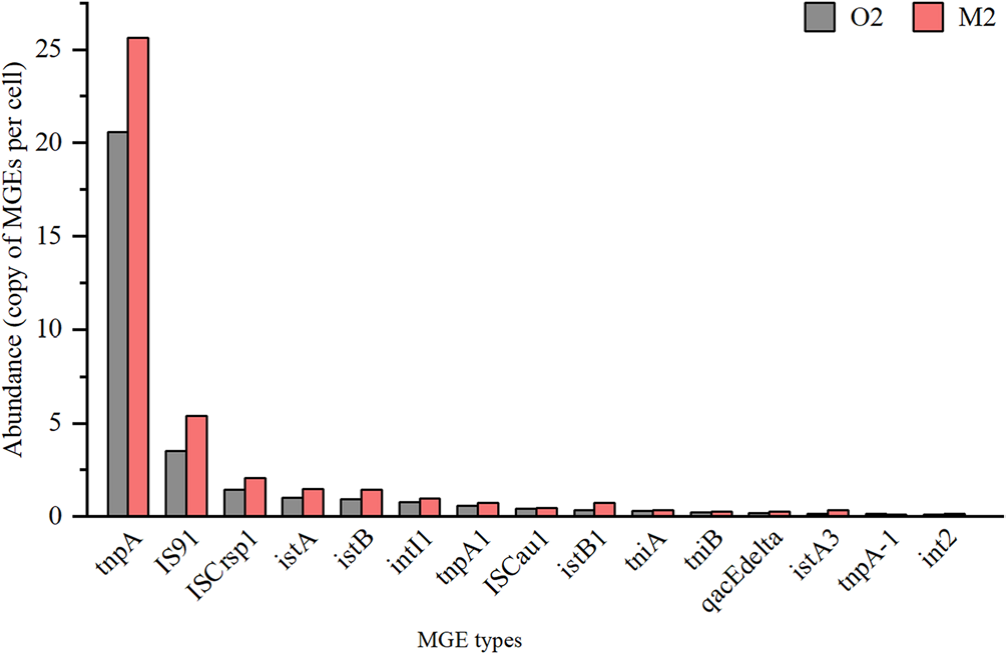
Distribution and relative abundance of the top 15 MGE types in the OD and MBR activated sludge samples.

Samples O2 and M2 harbored 119 and 122 MGEs, respectively. There were 100 MGEs of the same type shared between the two samples, 19 unique to O2 and 22 unique to M2. Figure 6 shows the top 15 MGEs in both samples, most of which represented plasmids, transposons, integrons and insertion sequences (ISs) and were more abundant in the MBR than in the OD. Among them, *tnpA* had the highest abundance at 20.60 and 25.66 copies of MGEs per cell, accounting for 63.32% and 59.76% of the total MGEs in the OD and MBR, respectively, followed by *IS91*, *ISCrsp1*, *istA*, *istB* and *intI1*.

## Discussion

A total of 24,041,065 bacterial sequences were detected, accounting for 99.61% of all microorganisms, and all of them could be classified into 29 phyla. The dominant phyla in all samples were Proteobacteria (43.24–63.69%), Actinobacteria (7.42–41.88%), Firmicutes (2.07–31.32%), Bacteroidetes (1.23–13.01%), Chloroflexi (0.02–5.40%), and Nitrospirae (0.01–4.17%). Previous literature has shown that the microflora in wastewater and activated sludge from WWTPs in the world are similar at the phylum level, including Proteobacteria, Firmicutes, Bacteroidetes, and Actinobacteria^5,7,25,26^. In this study, Proteobacteria was absolutely dominant in all samples, playing roles in denitrification, phosphorus removal and organic degradation^5^. Firmicutes (31.32%) and Bacteroidetes (13.01%) had higher abundances in the Inf sample, and the former decreased to 3.65% and 2.07% in samples O-Eff and M-Eff, respectively, while the latter decreased to 5.80% and 2.02%, respectively. It has been reported that Firmicutes is mainly responsible for the degradation of lignocellulose and hemicellulose^27^. Bacteroidetes is responsible for the decomposition of polymers and complex organic compounds, producing simple molecules that are easy to absorb, transform and utilize by other microorganisms^26^. Furthermore, Actinobacteria, Chloroflexi, and Nitrospirae were enriched in activated sludge and wastewater. Actinobacteria can decompose complex compounds and certain toxic compounds, and Chloroflexi plays an important role in the degradation of carbohydrates and cellular materials^11^. The existence of these bacteria makes a great contribution to the removal of pollutants.

The heatmap (Fig. 2) shows the distribution of bacterial genera in all samples. *Acidovorax* (17.55%) was the predominant bacteria in sample Inf. It is worth noting that *Acidovorax* is commonly found in WWTPs with high abundance and has been indicated to carry *ermB* and *blaTEM*^28^*.* Other major bacteria were *Acinetobacter* (11.96%), *Bacteroides* (6.97%), *Lactobacillus* (5.13%), *Bifidobacterium* (4.25%), *Escherichia* (3.71%), and *Prevotella* (3.63%). Among these genera, *Acinetobacter* and *Bacteroides* are considered potential pathogens, and are also thought to carry a variety of types of ARGs and be resistant to multiple antibiotics^18^. In addition, *Escherichia* and *Prevotella* are human conditional pathogens. Notably, *Lactobacillus* and *Bifidobacterium* are both beneficial microorganisms in the human body^29^. These results indicated that there were a large number of pathogens and potential hosts of ARGs in the influent water of WWTPs, which need to be removed to reduce or even eliminate the risk of ARG transmission.

The abundance of the main bacteria in the influent decreased to varying degrees in the effluent sample. The relative abundance of *Acidovorax* in samples O-Eff and M-Eff was reduced to 8.95% and 6.43%, respectively. Similarly, *Acinetobacter* and *Bacteroides* were reduced to 5.78% and 2.14% in O-Eff and 1.86% and 0.02% in M-Eff, respectively. *Escherichia* and *Prevotella* also clearly decreased in O-Eff. This result implies that the enrichment degree of ARG-carrying bacteria and pathogens in OD effluent was higher than that in MBR effluent, which may be due to the interception of the MBR membrane to prevent microorganisms from being washed out^30^. Although these dominant components in the influent decreased to some extent, they did not disappear completely, and some harmful bacteria even increased. In sample O-Eff, *Luteimonas* (5.91%), *Acinetobacter* (5.78%), *Mycolicibacterium* (4.70%), *Arcobacter* (4.47%), and *Tetrasphaera* (3.63%) were more abundant. Comparatively, *Luteimonas* (20.01%) and *Deinococcus* (13.62%) were the main bacteria in sample M-Eff, and *Tetrasphaera* (6.91%) and *Mycolicibacterium* (4.83%) also had higher abundances. *Luteimonas* has a strong ability to degrade starch and can be detected in biofilters, soil, sediment, fresh water and sea water^31^. *Deinococcus* is known for its ability to repair large amounts of DNA damage and is highly tolerant to extreme radiation and dry conditions^32^, and *Tetrasphaera* is a common phosphorus removal bacterium in WWTPs^33^. *Arcobacter* and *Mycolicibacterium* are not only pathogens of humans and animals but also potential hosts of ARGs and MGEs^18^. More specifically, *Mycolicibacterium* carries the *gyrA* gene and is resistant to fluoroquinolones^34^. These ARG-carrying pathogens can lead to the spread of disease and drug resistance^21^, which is worthy of attention.

In addition, there were other pathogenic bacteria with low abundance (0.09–1.30%) in both effluent samples, such as *Enterobacter*, *Escherichia*, and *Streptococcus*^18^. After processing treatment, there were still many kinds of pathogenic bacteria and microorganisms carrying ARGs in the effluent. A previous study on the removal efficiency of ARGs in wastewater by five processes (activated sludge, MBR, SBR, upflow anaerobic sludge blanket (UASB), and biological filter) showed that all of these processes can remove pathogens to some extent although there are still residues in the effluent^35^, which is similar to the results of this study. More efforts need to be made to study the inactivation of pathogens and ARGs in the effluent after ultraviolet disinfection.

For activated sludge, samples O1 and O2 contained mainly *Acidovorax* (16.03% and 9.23%), *Mycolicibacterium* (4.02% and 3.92%), *Candidatus_Promineofilum* (3.87% and 3.66%), *Micropruina* (3.75% and 3.59%), *Thauera* (3.23% and 3.89%), and *Nitrospira* (2.72% and 4.26%). Similarly, in both samples O2 and M2, the main bacteria were *Mycolicibacterium* (10.02% and 13.12%), *Tetrasphaera* (9.06% and 9.06%), *Acidovorax* (6.73% and 6.73%), *Candidatus_Promineofilum* (5.31% and 5.50%), and *Micropruina* (4.37% and 2.54%). This implies that the distribution of bacteria in the OD and MBR activated sludge was similar, and the clustering result of the heatmap (Fig. 2) also confirms this point. In addition, there was no significant difference in the relative abundance of bacteria between the two kinds of activated sludge (O1 and M1, and O2 and M2), by ANOVA (*p* > 0.05). A recent study indicated that similar wastewater treated by different processes (ODs, A^2^O and cyclic activated sludge system (CASS)) led to a similar microbial community distribution^18^. Another study on the treatment of municipal wastewater with AO and A^2^O also showed that the microbial communities in activated sludge were similar^36^. These results are consistent with the results of this study that under the same influent conditions, the microorganisms in activated sludge are not affected by the type of process.

Further analysis of the identified functional bacteria for nitrogen and phosphorus removal in activated sludge showed that *Nitrosomonas* (0.04–0.90%) and *Nitrospira* (1.48–3.73%) were the main AOB and NOB, respectively, which is in line with other WWTPs^18^. These two nitrifying bacteria grow slowly, and as typical "K-strategists", they have the advantage of using resources efficiently and growing at the longer SRT employed by MBRs^30^. This is similar to the results reported by Ma et al., in that *Nitrosomonas* (0.33%) and *Nitrospira* (0.17%) had higher abundances in the MBR process than in a conventional activated sludge process after treatment of the same wastewater^7^. Both *Tetrasphaera* and *Accumulibacter* are widely regarded as the main PAOs in activated sludge^37^. *Tetrasphaera* can rely on fermentation metabolism for cell maintenance and proliferation, and its fermentation products can even supply *Accumulibacter* with synergistic phosphorus removal^33^. In this study, *Tetrasphaera* was the most abundant phosphorus-accumulating bacteria in the two kinds of activated sludge, with a relative abundance of more than 2.79%, while *Accumulibacter* was less than 0.6%. A previous study suggested that *Tetrasphaera* can absorb hydrophilic substances under anaerobic, anoxic and aerobic conditions and accumulate hydrophobic substances, such as long-chain fatty acids, for long-term survival. In contrast, *Accumulibacter* can hardly survive under a long-term hypoxia/aerobic alternate environment^38,39^, which explains why the proportion of *Tetrasphaera* in most WWTPs is much higher than that of *Accumulibacter*^33^. The existence of these functional microorganisms provides a reasonable explanation for the good nitrogen and phosphorus removal efficiency of the OD and MBR processes. Surprisingly, these functional bacteria were confirmed to carry many types of ARGs, such as *Nitrosomonas* and *Nitrosospira* carrying *EF-TU*^16^; *Nitrosomonas* may also carry *penA*, *oqxBgb*, *vanHAc2*, *vanR-F*, and *dfrK*^17^; and *Accumulibacter* is a host of *peb-EC*, *SFO-1*, *vanR-B*, and *vanR-C*^17^. Although the relative abundance of these functional bacteria in the influent was very low (< 0.1%), it was detected in activated sludge and effluent and would pose a potential threat to the receiving environment.

Most ARGs entering the wastewater treatment system are carried by microorganisms^21^. Multidrug (27.31%), fluoroquinolones (12.97%) and polypeptides (12.91%) were the top ARG types identified in sample Inf. A study found that macrolides, tetracyclines, aminoglycosides, beta-lactams, and sulfonamides were the most abundant in untreated wastewater in 60 countries around the world^40^, which is significantly different from the results of this study and may be influenced by socioeconomic, health and environmental factors^40^. The ARGs in the activated sludge samples were consistent with those in the influent, namely, multidrug (24.91–25.57%), fluoroquinolones (13.14–14.40%) and polypeptides (12.46–13.10%), which is different from the other regions of China (Beijing^17^, Shanghai^16^ and Hong Kong^20^). It is worth noting that all major ARG types were consistent in type and similar in relative abundance in the OD and MBR. However, it can be seen from the effluent samples that the removal proportion of OD on multidrug is significantly higher than that of MBR, while the removal proportion of these two processes on other drug resistance categories were relatively similar.

Figure 5 clearly shows the relative abundance of the top 15 ARG subtypes in all samples. These ARGs exist throughout the WWTP, and there is a transition from the original influent to the final effluent^41^. The total relative abundance of ARGs in sample Inf was as high as 3617.47 ppm and was 791.34 ppm and 3774.37 ppm in samples O-Eff and M-Eff, respectively. Specifically, the relative abundances of *macB*, *bcrA*, *evgS*, *PmrA*, *desR*, and *efrA* clearly decreased in the O-Eff sample, while many ARGs, such as *mfd*, *rpoC*, *ala*, *bcrA*, and *murA*, increased in the M-Eff sample. The results show that the ARG removal effect of the OD process is better than that of the MBR process. This result is similar to the findings of Yuan et al., who noted that the activated sludge method greatly reduced ARGs, while MBR did not^35^. This result is surprising as most previous results have shown that MBR is the most effective way to remove ARGs from wastewater due to ARGs been trapped by the membrane. A large number of microorganisms were trapped in the MBR membrane pool; under the conditions of high aeration and stirring, the cells were lysed, and intracellular DNA (iDNA) leaked out, which may lead to the persistence of extracellular DNA (eDNA) in the effluent after MBR treatment^35^. In addition, the higher biomass concentration in sample M1 (expressed by suspended solids in mixed liquid (MLSS), 10,380 mg/L) than in sample O1 (4834 mg/L) also indicates that there will be more DNA leakage. The migration of eDNA is an important reason for the migration and transformation of ARGs. Current evidence shows that high activated sludge concentrations and biofilm- and antibiotic-resistant bacteria in the MBR process provide favorable conditions for horizontal transfer of ARGs (*Rp4* plasmid)^42^. The good settling performance of activated sludge may lead to the transfer of most bacteria to biological solids, which is helpful to reduce eDNA. Compared with sample O1 (94 mg/L), sample M1 (156 mg/L) has a higher sludge volume index (SVI) value, so the MBR process cannot effectively prevent the leakage and dissociation of eDNA. Previous studies have confirmed that a decrease in ARGs is positively correlated with a decrease in the microbial community (*p* < 0.01)^43^. Further study of eDNA is more convincing to the speculated results in the future. In addition, the pore size of the membrane is also one of the important factors affecting ARG removal^44^. The MBR process with an average pore size of 0.1–0.4 μm was used in a WWTP in Wuxi (China) and showed a notable removal effect on three tetracycline resistance genes (*tetG*, *tetW* and *tetX*), one sulfonamide resistance gene (*sul1*) and one class 1 integron gene (*intI1*)^43^.

Most of the ARGs in the influent are transferred to the activated sludge^19^. The distribution of ARGs in sludge samples was similar to that in Inf samples; *macB* was the most abundant, followed by *patA*, *mfd*, *rpoC*, and *oleC*. ANOVA showed that there was no significant difference in the abundance of ARGs between the two kinds of activated sludge (*p* > 0.05). Thus, it was inferred that the ARGs in activated sludge are not affected by the process when treating the same influent. Recently, a study showed that changes in microbial communities directly lead to changes in ARGs^17^. Relevant studies have also shown that bacterial abundance is an important factor affecting ARGs; the higher the abundance is, the greater the associated ARG abundance^2,18^. Direct evidence is provided by the fact that OD and MBR activated sludge have similar bacterial community distributions under the same influent conditions.

Most of the ARGs are located on MGEs, which makes it possible for microorganisms in the distant taxonomic lineage to obtain ARGs^45^. Plasmids are considered to be the main carriers of ARGs. Che et al. found that in addition to multidrug resistance, all other ARGs detected in three WWTPs in China included aminoglycosides, macrolide-lincosamide-streptogramin (MLS), β-lactam, tetracycline, chloramphenicol, quinolone and trimethoprim, which are mostly carried by plasmids and provide convenience for ARGs migration and transformation^46^. In this study, tetracycline (*tetQ*, *tetO* and *tetM*), beta-lactam (*CfxA6*, *CfxA3*), and MLS (*ErmF*, *ErmG*) were carried by integrative and conjugative elements (ICEs), which can lead to multiple resistance to a variety of gram-positive and gram-negative pathogens^47^. The ARGs on the chromosome cannot move themselves, and horizontal transfer can be achieved if they are integrated into MGEs^46^. Some ARGs, such as macrolide (*macB*), quinolone (*gyrA*), sulfonamide (*sul1*), tetracycline (*tetA*, *tetW*), *OXA-347*, and MLS (*ErmF*), were confirmed to be located on chromosomes. These ARGs detected in this study run through the whole WWTP, and they bring hidden dangers to human health and ecosystems with the migration and transformation of MGEs.

Furthermore, we identified the existence of MGEs, such as plasmids, transposons, integrons and ISs, which, as carriers of ARGs, play an important role in the horizontal transfer of ARGs^17^. Transposons and integrons are generally believed to be the main acquisition drivers of ARGs by MGEs^53^. Among them, *tnpA* and *intI1* are often regarded as important markers of MGEs and have frequently been found in different environmental media^48,49^. Quantitative analysis of detected MGEs found that the transposon *tnpA* was the most abundant, with 20.60 and 25.66 copies of MGEs per cell in sample O2 and sample M2, respectively. Li et al. suggested that *tnpA* is positively correlated with Bacteroidetes and MLSS^49^. In this study, Bacteroidetes in sample M2 (7.01%) was more abundant than that in sample O2 (4.01%), while the MLSS in sample M2 (9381 mg/L) was notably higher than that in sample O2 (4356 mg/L), which may be the reason that the abundance of *tnpA* in the MBR was higher. *intI1*, a common typical integron, also exhibits an intense positive correlation with MLSS^49^, which results in a slightly higher abundance in sample M2 (1.00 copies of MGEs per cell) than in sample O2 (0.80 copies of MGEs per cell). Other detected MGEs, such as *IS91*, *ISCrsp1*, *istA* and *istB*, had relatively high abundance. All of these are categorized as ISs, which are DNA fragments that can be transferred between different positions on the same chromosome or between different chromosomes and are highly diverse types of MGEs that are often detected in the activated sludge of WWTPs^17^. A significant positive correlation exists between ARGs and MGEs^50^, and these MGEs detected in the OD and MBR may lead to the propagation and transformation of ARGs.

## Materials and methods

### WWTP overview and sludge sample collection

The WWTP was located in Urumqi, Xinjiang, China. The first phase adopts an OD process followed by a deep bed denitrification filter with a treatment volume of 8 × 10^4^ m^3^/day. The second phase adopts an A^2^O + MBR process with a treatment volume of 12 × 10^4^ m^3^/day. The two processes treat the same influent, which is mainly domestic sewage. The inlet and outlet water parameters were measured for BOD_5_ (280 and 6 mg/L, respectively), COD (620 and 30 mg/L, respectively), SS (350 and 10 mg/L, respectively), NH_4_^+^-N (5 and 1.5 mg/L, respectively), TN (65 and 15 mg/L, respectively) and TP (7 and 0.3 mg/L, respectively). In order to strengthen the phosphorus removal effect, chemical agents were added at the back end of the OD process and the front end of the MBR process. The influent water was collected from the distribution well, the effluent water of the OD and MBR were collected from the outlet of the secondary sedimentation tank and the membrane tank, respectively, and activated sludge samples were collected from the aerobic sections of the OD and the MBR membrane tanks. Table 2 provides the particular sample acquisition information.

**Table 2:** Sample information collection.

Samples	T (℃)	DO (mg/L)	HRT (h)	SRT (d)	MLSS (mg/L)	SVI (mg/L)	Collection date
Inf	19.3	–	–	–	–	–	9/29/2020
O-Eff	19.1	–	–	–	–	–	
M-Eff	20.3	–	–	–	–	–	
O1	19.7	1.23	11.90	20	4834	94	
M1	20.6	6.98	1.58	> 20	10,380	156	
O2	18.2	1.40	12.30	20	4356	82	10/16/2019
M2	19.4	7.20	1.34	> 20	9381	158	

### Measurement of pollution indicators and activated sludge

BOD_5_, COD, SS, NH_4_^+^-N, TN and TP were monitored by standard methods. DO and water temperature were measured with online monitors. The concentration of MLSS was determined by the gravimetric method. The SVI value refers to the volume of 1 g of dry sludge after the aeration tank mixture settled for 30 min (calculated in terms of mL).

### DNA extraction, database construction and metagenomic sequencing

The E.Z.N.A. Soil DNA Kit (Omega Bio-Tek, Norcross, GA, USA) was used to extract DNA from all samples in accordance with the operational manual. The DNA integrity was then determined by 1% agarose gel electrophoresis. The concentration and purity of the DNA were detected using TBS-380 and NanoDrop200 fluorometers, respectively.

The DNA was broken into 400 bp fragments with the ultrasonic breaker Covaris M220 (Gene Company, China), and then the paired-end library was constructed by NEXTFLEX Rapid DNA-Seq (Bioo Scientific, Austin, TX, USA). First, the splice of the fragment was linked, and then the splice self-attachment fragment was removed by magnetic bead screening. Next, the library template was enriched by PCR amplification, and finally, the PCR product was recovered by magnetic beads to obtain the final library.

Metagenomic sequencing was performed on the Illumina NovaSeq/HiSeq Xten sequencing platform by Majorbio Bio-Pharmaceutical Technology Co., Ltd. (Shanghai, China). The original sequences obtained in this study were deposited in the Sequence Read Archive (SRA) database of National Center for Biotechnology Information (NCBI) under accession number SRP311506.

### Bioinformatic analysis

First, fastp^51^ was used to cut the 3′ and 5′ ends of the original adapter sequences. After removing the splicing, the length of the adapter sequence was less than 50 bp, the average base quality value was less than 20, and the retained reads, containing N bases, were high-quality paired-end reads and single-end reads. Next, MEGAHIT^52^ was used to assemble the optimized sequences, and contigs ≥ 300 bp were selected for final assembly. The assembled reads were used for further gene prediction and annotation. Open reading frame (ORF) prediction was performed with MetaGene^53^, and motifs ≥ 100 bp were selected and translated into amino acid sequences. A nonredundant gene catalog was constructed using CD-HIT^54^, and the identity and coverage were both 90%. Reads after quality control were mapped to the nonredundant gene catalog with 95% identity using SOAPaligner^55^, and the abundance information of the gene was counted in the corresponding sample. Taxonomic classification of metagenomes was analyzed by Kraken2^56^, and the relative abundance was estimated by Bracken^57^. The amino acid sequence of the nonredundant gene set was compared with the CARD database using DIAMOND (the BLASTP alignment parameter e-value was set to 1e^−5^), and then the corresponding annotation information of the antibiotic resistance function was obtained. Finally, the relative abundances of the ARGs were represented as ppm (i.e., sequences annotated as ARGs per one million sequences).

### Quantification of MGEs

The original sequences were processed for quality control by fastp (v0.12.1) using the default parameters. The resulting filtered reads were then used as the inputs for pipeline ARGs-OAP (v2.0)^58^, which consists of a SARG database and uses a reference MGE database to replace SARG (v2.0) for quantitative analysis of MGEs in the samples^60^. The reference MGE database^59^ contains 278 different annotated genes and more than 2000 unique sequences and is available at https://github.com/KatariinaParnanen/MobileGeneticElementDatabase. The number of MGE copies per cell was calculated by normalizing the abundance relative to the number of MGEs with MGE copies as the unit^58^.
